# Draft genome sequence of *Lacticaseibacillus paracasei* subsp. *paracasei* SCA72564 isolated from *Dioscorea esculenta* (Lour.) Burkill in Ilocos Norte, Philippines

**DOI:** 10.1128/mra.00480-26

**Published:** 2026-06-04

**Authors:** Marvielyn Porillo, Jan Ephraim R. Vallente, Lemuel Ray Balloyan, Jimmbeth Zenila Fabia, Shaira Dawn Oaquera, Doreen Domingo, Peter James Icalia-Gann

**Affiliations:** 1 Center for Cellular and Molecular Medical Research, College of Medicine, Mariano Marcos State University, Ilocos Norte, Philippines; 2 Research Directorate, Mariano Marcos State University Ilocos Norte, Ilocos Norte, Philippines; 3 Department of Biological Sciences, College of Arts and Sciences, Mariano Marcos State University, Ilocos Norte, Philippines; 4 Department of Biochemistry and Molecular Biochemistry, College of Medicine, Mariano Marcos State University, Ilocos Norte, Philippines; Nanchang University, Nanchang, Jiangxi, China

**Keywords:** probiotics, whole-genome sequence

## Abstract

Here, we report the draft genome sequence of *Lacticaseibacillus paracasei* subsp. *paracasei* SCA72564, a potential plant-derived probiotic isolated from *Dioscorea esculenta* tubers in the Philippines. The *de novo* assembly comprises 40 contigs, with a total length of 3.07 Mb, an N50 of 347,929 bp, and a GC content of 46.12%.

## ANNOUNCEMENT

The functional food industry’s dependence on dairy products limits accessibility for lactose-intolerant populations, highlighting the need for sustainable, plant-based probiotic alternatives (1). In Northern Philippines, probiotic strains have been isolated from underutilized indigenous plants, supporting their valorization as sources of beneficial microbes (2–5). The lesser yam, *Dioscorea esculenta* (Lour.) Burkill, possesses a rich phytochemical and prebiotic composition that provides a favorable niche for resilient lactic acid bacteria (6). Here, we report the draft genome of *Lacticaseibacillus paracasei* subsp. *paracasei* SCA72564 (7), isolated from *D. esculenta* tubers collected in Camandingan, Batac City, Philippines (18.0566° N; 120.5639° E), to evaluate its biotherapeutic safety and functional potential.

Tubers were peeled, diced, and rinsed with sterile distilled water and then incubated in de Man, Rogosa, and Sharpe (MRS) broth at 37°C for 4 days. The enrichment culture was streaked onto MRS agar and incubated at 37°C for 24–48 h. Colonies with typical lactic acid bacteria morphology were purified through repeated streaking. Pure isolates were preserved in 50% (vol/vol) glycerol at −80°C.

For genomic analysis, 1.5 mL of overnight culture grown in MRS broth at 37°C was centrifuged for DNA extraction using the GF-1 Bacterial DNA Extraction Kit (Vivantis, Malaysia) following the manufacturer’s protocols. Libraries were prepared with the Nextera XT DNA Library Preparation Kit (Illumina, USA) and sequenced on the Illumina MiSeq (Illumina, USA) platform with paired-end reads of 150 bp. SCA72564 sequencing data have a total of 7,906,254 reads, with the number of bases totaling to 1,190 Mbp. Raw reads were quality-checked using FastQC v0.12.1 (8), removal of NexteraPE adapters as well as leading/trailing clipping was done with Trimmomatic v0.40 (9) (Q < 20), and assembled *de novo* using SPAdes v4.2.0 (10) with the --careful option. Assembly quality was assessed using QUAST v5.3.0 (11), while completeness and contamination were evaluated with BUSCO v6.0.0 (12) and CheckM2 v1.1.0, respectively (13). Taxonomic identity was confirmed via the MiGA webserver (14), and annotation was performed using Bakta v1.12.0 (15). Functional potential was assessed through antiSMASH v8.0.4 (16) and gutSMASH v2.0.1 (17). *In silico* safety screening for antimicrobial resistance genes, virulence factors, and mobile genetic elements was conducted using CARD v4.0.2 (18), VirulenceFinder v2.0.4 (19), and PlasmidFinder v2.1.6 (20). ProbioMinServer2 (21) integrated these results to generate a Probiotic Potential Risk Score (PPRS). Default parameters were applied unless otherwise specified.

The draft genome comprised 40 contigs with a total length of 3.07 Mb, an N50 of 347,929 bp, and completeness score of 99.0% and 99.94% in BUSCO and CheckM2, respectively. Taxonomic analysis showed 98.08% average nucleotide identity (ANI) similarity to *L. paracasei* subsp. *paracasei* JCM 8130^T^. Annotation identified 2,952 genes, including 2,869 protein-coding sequences. No acquired antimicrobial resistance genes and transmissible virulence factors were detected based on the database used, supporting the strain’s favorable safety profile. Detailed assembly and annotation metrics are provided in Table 1.

**TABLE 1:** Assembly and annotation results of strain SCA72564

Assembly statistics	SCA72564
Number of contigs	40
Genome size (bp)	3,065,499
Largest contig (bp)	651,721
GC content (%)	46.12
N50 (bp)	347,929
L50	4
Coding density (%)	85.4
CheckM2 completeness	99.94%
BUSCO (lactobacillaceae_odb12)	99%
CDS	2,873
tRNA	57
rRNA	3
Probiotic Potential Risk Score	2.0

## Data Availability

The raw sequencing reads and the assembled whole-genome sequence have been deposited in the NCBI Sequence Read Archive (SRA) and GenBank, respectively, under BioProject accession number PRJNA1293312 and BioSample accession number SAMN53139918. The raw Illumina paired-end sequencing reads are accessible under SRA accession number SRR35991900. This Whole-Genome Shotgun project has been deposited in DDBJ/ENA/GenBank under accession no. JBSNBU010000000. The version described in this paper is the first version, JBSNBU010000000.
